# Paving the way to better understand the effects of prolonged spaceflight on operational performance and its neural bases

**DOI:** 10.1038/s41526-023-00295-y

**Published:** 2023-07-31

**Authors:** A. C. Stahn, D. Bucher, P. zu Eulenburg, P. Denise, N. Smith, F. Pagnini, O. White

**Affiliations:** 1 Unit of Experimental Psychiatry, Perelman School of Medicine at the University of Pennsylvania, Philadelphia, PA USA; 2 Charité—Universitätsmedizin Berlin, corporate member of Freie Universität Berlin and Humboldt-Universität zu Berlin, Institute of Physiology, Berlin, Germany; 3 IZN-Neurobiology, University of Heidelberg, Heidelberg, Germany; 4 Institute for Neuroradiology & German Center for Vertigo and Balance Disorders, Ludwig-Maximilians-University Munich, Munich, Germany; 5 Normandie Univ. UNICAEN, INSERM, COMETE, CYCERON, Caen, France; 6 Protective Security and Resilience Centre, Coventry University, Coventry, United Kingdom; 7 Department of Psychology, Università Cattolica del Sacro Cuore, Milan, Italy; 8 Université de Bourgogne INSERM-U1093 Cognition, Action, and Sensorimotor Plasticity, Dijon, France

**Keywords:** Neuroscience, Human behaviour

## Abstract

Space exploration objectives will soon move from low Earth orbit to distant destinations like Moon and Mars. The present work provides an up-to-date roadmap that identifies critical research gaps related to human behavior and performance in altered gravity and space. The roadmap summarizes (1) key neurobehavioral challenges associated with spaceflight, (2) the need to consider sex as a biological variable, (3) the use of integrative omics technologies to elucidate mechanisms underlying changes in the brain and behavior, and (4) the importance of understanding the neural representation of gravity throughout the brain and its multisensory processing. We then highlight the need for a variety of target-specific countermeasures, and a personalized administration schedule as two critical strategies for mitigating potentially adverse effects of spaceflight on the central nervous system and performance. We conclude with a summary of key priorities for the roadmaps of current and future space programs and stress the importance of new collaborative strategies across agencies and researchers for fostering an integrative cross- and transdisciplinary approach from cells, molecules to neural circuits and cognitive performance. Finally, we highlight that space research in neurocognitive science goes beyond monitoring and mitigating risks in astronauts but could also have significant benefits for the population on Earth.

## Introduction

**Fig. 1:**
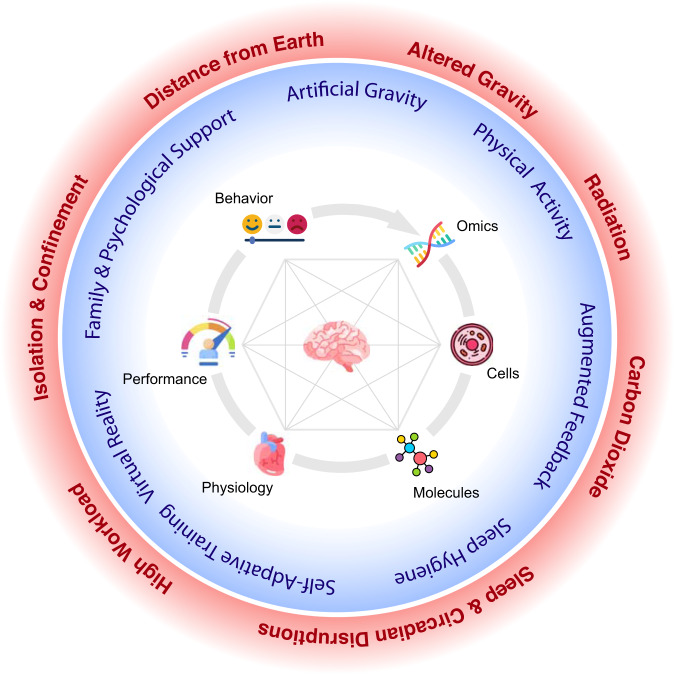
Integrative transdisciplinary research approach to systematically identify the crosstalk of manifestations between behavioral systems (physiologic, cognitive, and self-report) and their neural bases (genetic, molecular, and cellular). Red circle indicates environmental and operational stressors. Blue circle shows countermeasures to mitigate neurobehavioral risks. The countermeasures are expected to be synergistic and need to be targeted to individual needs (individualized countermeasures). The brain that orchestrates all processes is at the center of these concentric circles. Gray lines indicate the mutual interdependencies between omics, cells, molecules, integrative physiology, and their neurobehavioral signatures.

Future exploration class space expeditions will be some of the most difficult, dangerous, and dynamic operations in the history of mankind, ranging from Earth orbit operations to deep space exploration. They will push the limits of human performance and critically rely on the integrity of a range of organ systems and functions. With the advent of longer spaceflight missions space agencies have recognized the importance of better understanding the effects of environmental, operational, and psychological hazards during exploratory class missions on brain and behavior. The potential damage to neural structures in response to spaceflight and its behavioral implications are not well understood. At the same time adverse behavioral conditions, psychiatric disorders, and sensorimotor deficits are considered some of the most serious but also least understood risks during future long-duration space missions. Many other spaceflight risks are ranked lower or have been mitigated. In addition to the physiological effects of microgravity, the spacecraft setting can involve exposure to multiple environmental toxicants and operational stressors^1^ that all have the potential to affect neurobehavioral performance (Fig. 1). Behavioral health and performance are governed by an interplay between the central nervous system (CNS), the environment and individual phenotypes that is characterized by constantly updating and balancing genetic, molecular, cellular, physiological and cognitive demands and resources. The CNS, and particularly the brain, have emerged as a pivotal area of research because of their vulnerability to various spaceflight stressors. There is increasing evidence that spaceflight is associated with various adaptations of the brain, including intracranial fluid shifts, gray matter changes, white matter declines, and sensory reweighting and neural compensation^2^. However, the exact changes in brain structure and function and their meaning and mechanisms, as well as the role of mission duration, sex, and phenotypic vulnerabilities are unknown. Likewise, data on the implications of brain changes relative to behavioral and operational risks are lacking. Given that errors, misjudgments, and accidents may have serious consequences, and can lead to loss of expensive equipment and compromise mission success, identifying and predicting the effects of spaceflight on human performance and understanding their neural correlates and molecular fingerprints will be critical for successful future exploration class missions^3,4^.

This exercise has been incepted by the Science Department of ESA’s Human Spaceflight and Exploration Directorate. In 2020, ESA released a Call for Ideas for evolution of the SciSpacE Roadmaps in the post 2024 era. The overarching objective of the call was to engage the European research community in refining the focus areas for the SciSpacE Human Research Roadmaps, particularly for the post 2024 period. Following an initial screening of the materials submitted, ESA appointed experts across Europe to set up several working groups reflecting relevant organ systems and human research areas for spaceflight. Each working group was self-organized and expected to present its summary at a meeting organized by ESA in late 2020. In 2021 preliminary reports were published by ESA for a further community consultation and review. Pooling the combined expertise of ESA with the scientific community and stakeholders across Europe led to the identification of four major future roadmaps including Behavioral Health & Performance (including Neuroscience & Psychology), Human Physiology, Pharmacological Countermeasures, and Integrative Countermeasures. Leveraging the outcomes of previous similar exercises (e.g., THESEUS) as well as opening up the knowledge-building process to the scientific community across disciplines, it has been possible to clearly define gaps and risks that are currently considered highly critical but unmitigated in space science and technology. The process of creating the roadmaps has involved hundreds of individuals from the wider life science and engineering communities, ESA and international partners. This strategy allowed to maximize cross-disciplinary synergies (interactions between working groups were strongly encouraged as of the start of the project). The outcome of this work has been summarized in a series of papers presented by each working group. The current manuscript highlights the ESA SciSpaceE research gaps identified in the field of Neuroscience as part of the Behavioral Health & Performance Roadmap; the Psychology expert group provided additional feedback on some aspects at the boundary between neuroscience and psychology. In the following sections, we identify and discuss some key knowledge gaps related to neurobehavioral performance during human future exploration space missions. Next, we address potential countermeasures to mitigate these risks. We conclude with a summary of key priorities for the roadmaps of current and future space programs, and how addressing these knowledge gaps can benefit people and industrial applications on Earth.

## Key knowledge gaps

### Brain and behavior—which cognitive domains are affected?

There is now considerable evidence that spaceflight conditions can negatively affect the brain. Koppelmans and colleagues (2016) reported extensive volumetric gray matter decreases, including large areas covering the temporal and frontal poles and around the orbits in response to spaceflight^5^. The same research group also observed white structural disruption in areas critical for visuo-motor control and higher-order visuo-spatial processing after spaceflight^6^. Notably, other groups reported increases in white matter immediately after spaceflight that persisted seven months after return to Earth^7^. Robust effects have also been observed for ventricular volume expansions^8–13^, which did not return to preflight levels six months^9,13^ and even up to one year after landing^10^.

The implications of these changes on human performance and behavior are unclear. Some of the most robust effects have been found for postural control. Using task functional magnetic resonance imaging (MRI) Hupfeld and colleagues (2022) demonstrated widespread reductions in cortical deactivation in somatosensory and visual areas after spaceflight that were associated with postural changes in balance decline^14^. Likewise, it was also shown that spaceflight-induced changes in cortical thickness of vestibular and sensorimotor regions, including the posterior insula and the superior temporal gyrus, predicted postural balance performance^15^. The effects on cognitive performance (as compared to sensorimotor control) are less clear^16^. While various neurobehavioral effects have been observed in response to specific spaceflight stressors (e.g., sleep deprivation, hypoxia, hypercapnia and irradiation), most data collected during spaceflight show stability, or performance increases throughout the mission^17,18^. The inconclusive findings can at least somewhat be attributed to study designs and methodological limitations such as mission duration, the type of tasks to assess cognitive performance, and the number and spacing of data sampling time points. For instance, Moore and colleagues (2019) observed significant impairments in manual dexterity, degradation in manual tracking, during dual tasking, and a car driving simulator task immediately upon return from space^19^. Notably, the effects recovered quickly (3–5 days post-landing), and the authors attributed their findings to post-flight blunting in low-frequency (‘tilt’) otolith function in addition to fatigue and environmental factors unique to prolonged stays in a spacecraft (e.g., increased CO_2_ levels, altered day/night cycles, high workload, confined space)^19^. Such effects could have a profound impact on operator proficiency such as controling a vehicle during the exploration of novel terrains, or performing rendezvous and docking maneuvers. It may also be possible that certain effects are delayed, and are only detected after weeks to months. For example, the NASA Twin Study showed that year-long human spaceflight does not immediately affect cognitive performance but reveals some adverse effects 6 months after return to Earth^20^. The temporal dynamics of cognitive performance trajectories during and after spaceflight are still to be explored. Along the same lines it is unclear whether there is a dose-response relationship between neurobehavioral adaptations and mission duration. It is therefore critical that future studies will go beyond 6-month duration missions, and consider following up space crews throughout the recovery of plus one year. Previous cognitive assessments also seem to have been driven by classical neuropsychological test paradigms. It is possible that spaceflight would benefit from more complex tasks that are specifically targeted to the astronaut population, and capture cognitive domains associated with visuo-spatial processing that are expected to be tightly coupled with operational tasks^5,6^. Moreover, it is expected that such tasks could be relevantly affected by gravitational changes because its close link to the vestibular system, which goes beyond maintaining gaze and balance. Interactions between the otoliths and semicircular canals critically contribute to spatial perception including self-motion, orientation, and navigation. It is possible that weightlessness affects various visuo-spatial abilities because of reduced gravitational stimulation. For example, it is known that spaceflight causes changes various vestibular responses leading to orientation illusions, sensory localization errors, and changes in vestibulo-ocular reflexes^21^. Cognitive tasks involving the neurovestibular system such as mental rotation^22^, perceived orientation^23^, and distance judgments^24^ are also affected during spaceflight. Some astronauts have reported a "time compression syndrome" in orbit^25^. They also require more time than normal to perform standard mental activity^26,27^. Previous microgravity research has also demonstrated the effects of weightlessness on posture, gaze, functional mobility, and spatial orientation, reporting misperceptions of visual orientation, depth and distance, and difficulties in shape recognition^28^. It is expected that any impairments in visuo-spatial abilities will also have adverse effects on spatial navigation performance. Given that the perception of self-motion depends on integrating visual information with gravitational cues processed by the vestibular system, spatial updating deficits are expected when gravity levels change. Data from parabolic flights though show that both micro- and hypergravity can lead to less precise spatial updating, a fundamental ability underlying spatial navigation^29^. This prediction is also in line with emerging evidence highlighting the cortical projections of the vestibular system.

Given the pivotal role of visuo-spatial abilities and time evaluation for mapping spatial relationships and operations such as docking, landing, and exploring and navigating in new environments and on planets with low gravity, it is imperative to understand the impact of spaceflight on spatial cognition and its neural basis. One of the key brain areas for mapping spatial relationships and performing navigational tasks is the hippocampus^30^. Animal data suggest that the hippocampus and related brain structures (i.e., parahippocampal, entorhinal, and perirhinal cortices) could be particularly vulnerable to the stressors associated with long-duration spaceflight such as radiation^31^, increased CO_2_ levels^32^, vestibular dysfunction^33^, stress^34^, sleep deprivation^35^, and isolation and confinement^36,37^. The effects of prolonged isolation and confinement on hippocampal plasticity have been demonstrated in humans, revealing significant reductions in dentate gyrus volume which were associated with reductions in key neurotrophins and adverse cognitive effects^38^. It is possible that the physiological effects of microgravity, environmental toxicants, and operational stressors interact and aggravate the consequences on brain plasticity during long-duration space missions.

### Sex as a biological variable

It is also unclear whether neurobehavioral differences in response to spaceflight are characterized by sex- and genetic-specific adaptations. For instance, on Earth there is little doubt that spatial navigation and its neural circuitry are characterized by sex-specific differences [e.g.,^39–41^]. It is unclear whether such differences also exist in astronauts, and if and to what extent spatial navigation and related brain changes are differently affected after spaceflight in men and women. In fact, our current understanding of neurobiological sex differences associated with spaceflight is critically limited because of the significant disproportion of sexes enrolled in previous astronaut corps. According to our best knowledge there are currently no data comparing brain changes between male and female astronauts. Elucidating the effect of sex is mandatory to fully understand brain plasticity and behavior and their molecular mechanisms^42,43^. This notion is in line with the world’s leading research centers to consider sex as a biological variable^44^, and the space agencies’ goal to increasingly strengthen the role of women in future space expeditions. For instance, the newest class of NASA astronaut candidates included almost 50% women^45^. In 2020 astronaut Christina Koch broke the record for the single longest spaceflight by a woman. With 328 days in space, she is among the top five all-time records for the longest single spaceflight. NASA also announced that the next person on Moon and the first person on Mars will likely be a woman^46^. Due to the increasing and essential role of women in future space missions, studies of both men and women are critically needed to better understand sex-dependent vulnerabilities to adverse neurobehavioral effects during future exploratory space expeditions such as a mission to Mars.

### Neurobiology

A central tenet of neuroscience is the cellular understanding of nervous system functions. Although great strides in quantifying the effects of spaceflight on neuronal physiology have been made, and despite the numerous constraints of space research^47^, a more synoptic understanding of the effects of altered gravity, space radiation, and prolonged spaceflight on the morphology and physiology of the various cell types of the nervous system and their synapses will be important to the success of future long-duration missions to the Moon or Mars. Morphological and functional alterations at the synapse could result in physiological impairments which fundamentally alter information processing in the nervous system. A more comprehensive understanding of spaceflight-induced physiological and structural alterations at the synapse and synaptic active zones, as well as the resulting changes in short and long-term plasticity and neurotransmitter homeostasis, are just a few of the relevant topics that need to be addressed in the years to come. These questions are not just relevant to glutamatergic, GABAergic, and cholinergic transmission but should be investigated in all relevant neurotransmitter systems (adenosinergic, glycinergic, histaminergic, monoaminergic) as well as the neuropeptides and gaseous signaling molecules.

Details regarding the effects of spaceflight on the nervous system have been poorly described so far. Animal experiments offer great potential to better understand the mechanisms underlying the neuronal responses to spaceflight. Spaceflight can significantly affect neuronal morphology and clearance of neuronal trash, highlighting the need to carefully assess the risks of long-duration spaceflight on the nervous system. Micro- and hypergravity responsive gene signatures identified several candidate targets with terrestrial roles in neuronal function and/or cellular metabolism, which are linked to regulation by daf-16/FOXO signaling. This could help to understand the basis of spaceflight-induced maladaptation.

Given the sparse availability of samples, the use of integrative omics technologies in multidimensional longitudinal studies to quantify metabolites, gene expression, and DNA methylation as well as DNA, RNA, protein, and chromatin dynamics after exposure to long-duration spaceflight will be paramount for furthering the understanding of space induced changes in cellular neurobiology^20^.

### Internal representation of gravity

Future crewed missions to the Moon and Mars will expose astronauts to varying levels of gravity. The neurophysiological responses to changing hypogravity (i.e., between 0 and 1 g) and hypergravity levels (i.e., > 1 g) are poorly understood. Gravity plays a fundamental role in human behavior and performance by providing critical cues to the vestibular system (see also discussion above) and supporting the concept of verticality (which way is “up” or “down”). The otolith organs detect linear, i.e., inertial and gravitational, accelerations acting on our bodies^48–50^. In microgravity, the gravitational force vector, which is predominantly sensed via the otolith organs (saccule > utricle), no longer acts as a sensory reference. The resulting mismatch between the vestibular, and the visual, proprioceptive and other sensorimotor signals can affect spatial orientation, hand-eye coordination, vertical but not horizontal oculomotor behavior, postural control and self-motion perception (for a comprehensive overview see Clément et al., 2020)^51^. The earliest symptom for this sensory mismatch phenomenon and modulation of otolith function is the elevated susceptibility for motion sickness during parabolic flight as well as during the first days of exposure to microgravity and upon return to Earth after a long-duration space mission^52^. This is most likely an adaptation process associated with the altered integration and interaction of information from the semicircular canals and the otolith organs. It is known that spaceflight causes various changes in several vestibular downstream responses including transient core level modulations in vestibulo-ocular reflexes^21^ leading to room tilt illusions and sensory localization errors. When tasks are performed during or shortly after any gravitational transitions sudden head movements especially in the yaw plane are not advised, further highlighting the role of canal-otolith integration.

Gravitational transitions can also induce aftereffects that will persist for some time after as the neurovestibular systems adapt to the new gravito-inertial loading. These neurological phenomena have been shown to impair behavior and performance across dimensions. While many studies have demonstrated that humans and animals eventually adapt to new gravitational environments (mostly microgravity)^53^, only a few have addressed the re-adaptation processes following re-entry: astronauts also experience a condition called “entry motion sickness”, which slows the speed of decision making and alters their ability to control the vehicle and their movements^54^. Neurovestibular challenges that occur when the crew member returns to normal gravity include alterations in manual control^20^, inability to exit the vehicle^55^, postural imbalance^56^, and locomotion disorders^57^. The first three weeks in space and the first two weeks back on Earth present critical adaptation periods that are characterized by impairments in perceptual-motor skills and higher attentional processes. However, the dynamics of re-adaptation when transitioning back to the original environment (e.g., 1 g) or to a new gravity level (e.g., Moon or Mars) is not well understood. This lack of knowledge may be partially explained by current methodological limitations and operational constraints; it is extremely challenging to collect data during or upon entry in a new environment or immediately after landing. The quantification of the dynamics of sensory processes with a short time constant (e.g., fast motor (re-)adaptation) requires accurate measurements immediately before and after entry, landing, or any other event that induces significant changes in the environment. Consequently, current methods of preflight training and post-flight rehabilitation have not been optimized to minimize the functional impacts of these natural adaptive responses during gravitational transitions or to restore environment-appropriate sensorimotor functions after these transitions.

Current research suggests a multimodal representation of gravity^58^, in which proprioceptive, somatosensory, visual and vestibular signals are integrated for processing gravity-related cues. For instance, Mittelstaedt (1996) proposed a somatic component for graviception that is located in the trunk, and particularly in the kidneys^59^. Humans use an internal model to dissociate gravity and inertial acceleration from a composite acceleration signal^49,60^. As a result of the complex integration of graviception and its interaction with motor control, understanding how the nervous system adapts to changes in gravity is challenging.

The precise nature and properties of this internal multimodal representation going beyond the vestibular system during acute gravitational transitions are also still to be determined. Experiments performed under altered gravitational levels have identified a rapid adaptation process. At the same time the data have also revealed biases indicative of partial habituation that could be observed throughout the entire (often short-term) exposure to weightlessness^61–63^. Early access to astronauts (immediately upon entry or after landing) is critical to characterize the dynamics of this highly dynamic adaptation process (see also previous section). This point has been emphasized in a previous white paper by White et al. (2016)^3^, and remains a critical issue. The next steps to deepen our understanding of the neural bases of adaptation to changes in gravity will require to address current methodological constraints such as technologies to measure brain function of cortical and sub-cortical structures in real-time. Furthermore, the modularity of dedicated discrete sensory organs and brain regions for processing gravity cues suggest a distributed role of structures and functions for perception and control^50^. In addition to visual, vestibular and internal (prior) information, somatosensory (proprioceptive) feedback and its influence on planning and control may serve as a calibration signal that is conditioning our perception and the scaling of motor commands. The importance of this sensory information has not been fully acknowledged so far. Finally, the experimental context itself faces several methodological challenges. Structural and functional magnetic resonance imaging (MRI) of the brain cannot be used to acquire data during gravity transitions. Data collected before and after an interventions (e.g., parabolic flight or centrifugation) can probe neural adaptations in response to acute exposures to altered gravitational levels. Yet, they must be cautiously interpreted because they do not allow to draw any conclusions about the effects of gravity levels and transitions. Although there is solid evidence of the existence of a vestibular network that processes gravity signals^50,64^, current approaches are insufficient to draw clear-cut conclusions, firstly for methodological reasons, and secondly because gravity in essence influences all systems at the same time. Technologies to observe brain function such as electroencephalography (EEG) and functional near-infrared spectroscopy (fNIRS) are mobile and could potentially be used to capture cortical neurophysiological changes in response to altered gravity in real-time. EEG records electrocortical activity at the scalp surface with temporal resolution and moderate spatial resolution (recent systems can support a density of 256 electrodes improving the accuracy of source localization techniques). In contrast, (f)NIRS reflects brain activation by measuring brain hemodynamic responses at lower temporal but higher spatial resolutions. Combining EEG and fNIRS may therefore overcome some of the previous methodological challenges and open new avenues to pinpoint how the brain creates an internal representation of gravity^65,66^. However, such approaches require cautious validation studies because of the potential confounding associated with cephalic fluid shifts on functional brain recordings^67^.

## Neurobehavioral countermeasures

Given the variety of environmental and psychological stressors associated with prolonged exploratory class missions, a combination of different treatments and interventions will be needed to mitigate neurobehavioral risks, maximize performance, and ensure astronaut health and safety, and mission success. Considering that responses to different spaceflight stressors are expected to vary between astronauts and their phenotypic differences, the strategies for neurobehavioral risk mitigation need to be personalized in terms of type, frequency, and intensity throughout a space mission. We here summarize some of the current and future approaches to maintaining neurobehavioral health and performance.

### Artificial gravity—a holistic approach that could also benefit brain and behavior?

Long-duration stay in microgravity on the various space stations demonstrated how gravity, or the lack thereof, has a significant impact on all organ systems such as bone decalcification, muscle loss, cardiovascular deconditioning, orthostatic intolerance, visual impairment, suppression of the immune system and other changes indicative of premature aging. Previous countermeasures to mitigate the effects of weightlessness have addressed different organ systems using target-specific approaches, i.e., lower body negative pressure^68^ and fluid loading to reduce orthostatic intolerance; resistive exercise and nutritional interventions to minimize muscle and bone loss; or aerobic exercise to maintain cardiorespiratory fitness^69^.

Delivering Earth-like gravity levels using artificial gravity (AG) could provide a holistic approach by mitigating physiological risks associated with prolonged exposure to weightlessness across all organ systems^70^. The idea of using AG to compensate for the negative effects of weightlessness is as old as spaceflight itself and has been considered a standard against which other countermeasures should be measured^71^. A large, ground-based, rotating platform that could house human subjects for long periods (weeks to months) while exposing them 24/7 to higher-than-normal gravity levels would offer a unique means to validate the long-term beneficial effects of small hypergravity on the human body across all organ systems^72^. Whereas the idea of generating AG by means of rotating parts of a spacecraft is compelling, rotation can have a number of undesirable side effects on performance^73^. For example, consider the Orion spacecraft which is about 5 m in diameter. To generate a force of about 1 g in a circular space with a 2.5-m radius, a rotation rate of 20 rpm would be required to generate a force of about 1 g. Depending on the location relative to the center of rotation body weight (gravitational gradients) changes. Likewise, walking in the direction of rotation will increase the centripetal force, and hence AG, and therefore also increase body weight^73^. In addition, such rotation speeds create significant Coriolis forces generated by body movements. To systematically study such effects DiZio and Lackner established a unique rotating chamber at the Ashton Graybiel Spatial Orientation Laboratory. This research resulted in a series of seminal studies on how lateral Coriolis forces can disrupt body movements and object transport and manipulation, how reaching errors carry-over in the opposite direction when the rotation stops, and that the human body can adapt to the Coriolis force and compensate the perturbations very rapidly^73^.

However, data on the long-term benefits of prolonged use of rotating chambers on the human body are lacking. In addition, the design and technical implementation of AG using rotating chambers in spacecrafts remains a considerable challenge. In contrast to the continuous rotation of a spacecraft to mimic Earth’s gravity, an efficient comprise could be the use of a short-arm radius centrifuge^74^. Exposing crewmembers to AG of 1 to 2 g at the heart for 0.5 to 2 h has been shown to be a promising countermeasure for reducing the effects on body unloading and fluid shifts^75^. AG has been shown to mitigate cardiovascular deconditioning^74,76–79^; muscle and bone loss^80–82^; improve neuromotor reflex function^83^; decrease headache severity associated with head-down tilt bedrest^84^; and alleviate some of the conditions of the spaceflight associated Neuro-Ocular Syndrome (SANS)^75^. AG also provides critical cues to the vestibular system. This is important because the vestibular system goes beyond maintaining gaze and postural stabilization. There is increasing evidence indicating that the vestibular system plays a critical role in brain plasticity and cognitive performance such as learning and memory formation^33^. For instance, patients with bilateral vestibular loss exhibited bilateral atrophy of the hippocampus of nearly 20%, which was correlated with spatial memory deficits in a virtual Morris water maze task^85^. The manipulation of the vestibular system has been shown to impair firing patterns of head direction cells (which are located in various brain areas including the subiculum, thalamus, and retrosplenial cortex^86^), hippocampal function, and medial entorhinal cortex^87–89^. Together these data highlight that vestibular stimulation provides critical cues for brain health and cognitive performance, supporting the notion that AG as a countermeasure could go beyond the musculoskeletal and cardiovascular system and also benefit brain and behavioral health. In addition, recent cell experiments suggest that hypergravity can have profound beneficial effects on neuronal health that are independent of vestibular stimulation. For instance, Lichterfeld et al. (2022) recently showed that hypergravity attenuates astrocytic reactivity, which is critical to limiting severe astrogliosis and the formation of the glial scar^90^. Likewise, exposure of murine primary hippocampal neurons to 2 g hypergravity increased neurite number by 30% and neurite projection length by 20%. In addition, mature synaptic contacts were formed under hypergravity conditions in neurons of later developmental stages^91^. These data provide evidence that hypergravity ameliorates neuronal cell growth and synaptic plasticity in vivo. Whether the effects of hypergravity on the cellular level also translate to humans is unclear.

Recent advances by space agencies are attempting to unravel the effects of AG on all organ systems including brain and behavior and to identify the most efficient protocols for using AG as a countermeasure. For instance, the NASA/ESA AGBRESA bed rest study carried out at DLR in 2019/2020 aimed to compare intermittent vs continuous AG exposures. Currently, ESA is carrying out two long-term bed rest studies investigating the effects of AG with different types of physical exercise. Specifically, the concept involves a hybrid approach of AG and physical exercise, i.e., exposing subjects to physical exercise while they are being centrifuged. Given the benefits of exercise across all organ systems, it is hypothesized that AG combined with physical exercise will interact in synergistically. With respect to the mitigation of neurobehavioral risks, it is noteworthy that physical exercise is also a potent stimulus for structural and functional brain plasticity^92^. Animal studies demonstrate that voluntary running increases synaptic plasticity and neurogenesis in the hippocampus^93^. The causal link between physical activity and hippocampal plasticity and cognitive benefits such as spatial memory performance has also been reported in a number of human studies^94–96^. The selective effects of AG and exercise on the brain suggest that the integrative approach of AG combined with exercise could go beyond affecting the cardiovascular and musculoskeletal system and also mitigate neurobehavioral risks associated with long-duration spaceflight in a highly efficient manner. While the hybrid approach of combining AG with physical exercise is promising it remains to be determined whether the benefits will go beyond exercise (or AG) per se and whether future crew vehicles will allow supporting the space and infrastructure of SAHC.

### Operational performance

Future long-duration space expeditions will be among the most difficult, dangerous and complex operations in history. Astronauts will be required to stay physically and socially isolated in confined spaces for 30 months, and longer. It is expected that not all skills and knowledge required for these missions can be retained and retrieved based on pre-mission training alone. Limited and delayed communication will significantly constrain support from Mission Control and crews will increasingly rely on autonomous onboard technologies to successfully perform rendezvous maneuvers, and telerobotic operations post-landing. Augmented feedback and autonomous self-adaptative, just-in-time training of spaceflight-relevant tasks may help to maintain operational performance.

### Autonomous self-adaptative, just-in-time sensorimotor training

Adaptive, just-in-time training relevant to these specific requirements could address this need by delivering tasks that adjust the difficulty level to the individual astronaut’s knowledge and skill learning curve. To mitigate this risk, research needs to identify the neural bases of key operational tasks, and design neuroscience-based adaptive visuomotor training programs that can be flexibly adapted to specific mission requirements and crew demands. These approaches should be characterized by (1) consolidating and improving sensorimotor skills relevant for inflight and post-landing operational tasks; (2) featuring an autonomous and adaptive training approach that does not rely on feedback from flight operations on ground; (3) maximizing the transfer of mission-relevant motor skills; (4) allowing the assessment of the neural circuitry underlying the task; and (5) delivering the training in a motivating and meaningful way to astronauts.

### Augmented feedback

How can human motor skills for operating human-machine interfaces such as controlers be maintained when gravitational levels change? Can fine skills be supported by manipulating vestibular and visual cues and controlling somatosensory and haptic information? Does the use of 1g-dynamics at the human/machine interface during altered gravity promote skill acquistion? Or would such feedback even impair learning and adaptation processes due to conflicting sensory information? Addressing these questions is complex because graviception and the internal representation of gravity is an integrative multimodal process. The basic rationale for using augmented feedback to maintain operational performance is that the central nervous system masters the motor control of complex tasks in 1 g. Delivering controler dynamics mimicking 1 g levels and/or providing haptic feedback could be expected to speed up learning and maintaining operational tasks. A few studies tested whether the simulation of a local terrestrial gravity (1 g) applied at the level of the hand improves pointing performance during altered gravity (0 g and 1.8 g)^97,98^. The data of this work are promising, showing pointing performance improvements and supporting the idea to design human-machine interfaces leveraging haptic feedback^98^. The effects of a simulation of weightlessness (0 g) and hypergravity (1.8 g) on the same task parameters have also been investigated on ground (i.e., 1 g). Remarkably, the simulation of gravitational fields different from 1 g applied locally to the effector produced effects that were comparable to immersion in an altered gravitational field^99^. These data demonstrate that the application of simple focal forces is sufficient to reproduce the effects of gravitational changes on relevant task parameters. In other words, more complex systems that simulate distributed force fields on the body, e.g., the upper arm, are not necessary. Together, the results underline the importance of the proprioceptive signals for the encoding of gravity, and also call for future research to better understand these effects with potential applications in robotic rehabilitation.

Finally, noise, like gravity, is also omnipresent in any environment and cannot be completed removed in the chain of physiological signal processing. Counter-intuitively, noise—or uncertainty in sensory signals—can be beneficial in some conditions. Stochastic resonance is a mechanism whereby a particular level of noise enhances the response of nonlinear systems to weak sensory signals. The effects of stochastic resonance on sensory modalities and, more particularly on somatosensory information have been demonstrated, paving the way toward its potential for improving sensorimotor performance as well as cognitive and autonomic functions^100^. These promising results demonstrate that stochastic resonance represents a flexible and non-invasive technique that can be applied to a variety of scenarios beyond astronauts, including in ambulant elderly, skilled movements, sports, and to patients with sensorimotor or autonomic dysfunctions^101^.

Future research on the use of self-adaptive, automous operational training and augmented feedback will help to better understand the fundamentals of fine motor skill acquisition and adaptation, and provide insightful information on the optimal design and control of human-machine interfaces and wearable robots in space environments and other immersive dynamics.

### Individualized countermeasures

The variety and specificity of neurobehavioral hazards and risks associated with spaceflight highlight the need for a combination of different approaches and methodologies to mitigate environmental and psychological stressors associated with prolonged space missions. These interventions comprise a range of methodologies, technologies, and approaches such as: self-adaptive training systems and augmented feedback to maintain operational performance; exercise and relaxation programs; schedules supporting sleep hygiene and maintaining regular work/rest cycles; engaging in meaningful work and learning; food, plant growth, VR sensory stimulation and entertainment; team building; family support; psychological counseling; balancing social and personal space; and habitat characteristics promoting health and well-being. Like personalized medicine, the administration of these measures should be based on the crewmembers’ individual needs instead of facilitating a single “one fits all” strategy applied to every crew member. Furthermore, the combination and prioritization of countermeasures should also be considered a dynamic process that varies within individuals and across time. In other words, the relative importance of specific strategies will vary during the course of a mission, requiring a constant reevaluation of the crewmembers’ individual needs. Stahn & Kühn (2021) referred to this concept as “Individualized Countermeasures” (ICount), which defines countermeasures as a dynamic construct that is optimized relative to the individual requirements as a function of mission duration. It requires the development of a systematic “toolbox” that combines a multiplicity of methodologies and approaches that can be flexibly utilized to meet crews’ individual needs^102^. The variety of approaches, the consideration of phenotypic differences, and the dynamic nature of individual preferences for specific needs during long-duration expeditions will be critical to maximize the benefits and synergies of neurobehavioral countermeasures.

## Priorities for the space program (microgravity and/or exploration relevance)

### An integrative framework from omics to behavior

Future long-duration space missions will be considerably longer than current 6-month missions on ISS. Mitigating cognitive and behavioral risks during such missions is a high priority for successful mission completion requiring several strategies that are summarized in Box 1. First, to address this gap research should target an integrative approach combining state-of-the-art immunological, neuroendrinological, behavioral, and imaging technologies that go beyond current experiments on ISS and spaceflight analogs. Multi-model neuroimaging approaches, including functional imaging to identify differences in brain networks and their connectivities, high-resolution structural imaging allowing to segment tiny, but critical subcortical brain areas such as the hippocampus with precision, and techniques to examine the role of the cortical circuitry associated with critical mission tasks control will play an important role to fully understand brain changes in response to spaceflight. This approach needs to go beyond standard clinical brain imaging but use novel imaging techniques such as multi-parameter mapping and include task functional MRI sequences that have high operational relevance, i.e., comprise tasks that allow to quantify the neural signatures of operational tasks such as docking and rendezvous maneuvers. In addition to leveraging operational performance tasks, cognitive testing should go beyond classical standard paradigms and neuropsychological test batteries. Future work should consider innovative tasks using a hypothesis-driven approach that is specifically targeted at the astronaut population relative to task type, difficulty, and duration. To account for effects related to the pathways between the vestibular signal processing, the limbic system and cortex imaging and behavioral measures should be linked to data from postural and functional mobility tests, videonystagmography, and vestibular evoked myogenic potentials. Given the importance of mood, sleep and circadian rhythms on cognitive function, they need to be considered as potential moderators of neurobehavioral performance. Exploring the relationships between neurobehavioral data and ocular examinations such as visual acuity, intraocular pressure, retinal nerve fiber layer thickness, and peripapillary and macular retinal thicknesses will allow corroborating the role of any visual impairments in response to spaceflight on cognitive performance. Key neurotrophins, oxidative stress markers, and neuroprotective cytokines are critical for brain health and behavior and have been shown to be modulated by physical (in)activity and spaceflight stressors. Biochemical assessments and advances in multi-omics technologies such as genomics, transcriptomics, proteomics, and metabolomics can therefore help to close knowledge gaps of the underlying molecular mechanisms and genetic drivers of neurobehavioral adaptations. Understanding the effect of sex and other phenotypic differences on sensorimotor and neurobehavioral changes is imperative because women will play an increasingly important role in future space missions. The overall approach should leverage the National Institutes of Mental Health (NIMH) Research Domain Criteria (RDoC) heuristic framework to integrate various levels of information from omics (e.g., genomics, transcriptomics, proteomics, metabolomics), cells, and molecular signatures to integrative physiology, neuroimaging, cognitive tests, and self-report. Such an integrative framework will allow us to better understand the basic neurophysiological dimensions of functioning underlying the full range of human behavior from normal to abnormal that relate to individual and interpersonal adaptation and vulnerability during long-duration spaceflight, and promote the development of effective, target-specific, and individualized countermeasures (Fig. 1). This line of research will build on current gaps in the present knowledge of this topic by delivering novel and unique data on behavioral, sensorimotor, and operational performance outcomes, and their neural basis. To this aim, the knowledge, technologies, and tools derived from such data relevantly contribute to the space agencies’ goal to provide knowledge, technologies, and tools to enable safe, reliable, and productive human space exploration. This will support mission planners and system developers with strategies for monitoring and mitigating crew health and performance threats that are currently unaddressed and considered high risks during future exploratory space missions.

Together, the combination of various levels of knowledge, methodologies, and outcomes highlighted here could provide the basis to better understand and characterize the type, extent, cause, and mechanisms of neurobehavioral changes in response to spaceflight and their phenotypic signatures. The complexity, expertise, effort, and costs cannot be leveraged by a single experiment. However, the way spaceflight and spaceflight analog studies are typically conceptualized has already the potential to address such a framework. Space agencies solicit announcements of opportunities to propose specific experiments. Following peer-review, feasibility assessment, and a definition phase, a final protocol integrates several experiments that typically address a range of physiological and behavioral research questions, including but not limited to cardiovascular, musculoskeletal, vestibular, immunological, and neurobehavioral outcomes. It is intriguing that researchers and agencies could leverage the integrative nature of such platforms and projects. Such an approach goes beyond data use agreements between experiments but starts with the identification of deliverables using a hypothesis-driven rationale that promotes a holistic understanding of intellectual frameworks that together exceed the individual disciplinary perspectives.

Box 1 Summary of key recommendations for mitigating operational performance deficits during exploration class missions

- Promote an integrative research framework that allows to collect data across neural and behavioral dimensions that maximize the understanding of cognitive and operational performance
- Understand sex as a biological variable: include the number of female astronauts being studied
- Better understand the internal representation of gravity, and its role in maintaining visuo-spatial abilities during changing gravity conditions
- Investigate the effects of spaceflight on spatial cognition and its neural basis
- Establish brain imaging that goes beyond the identification of clinical manifestations, e.g., task functional MRI sequences that have high operational relevance and allow to quantify the neural basis of cognitive impairments, and predict future performance in navigation tasks, docking maneuvers, and other spaceflight-relevant behavioral measures.
- Develop cognitive tasks using a hypothesis-driven approach that are specifically targeted at the astronaut population and expected to be particularly vulnerable to spaceflight
- Enhance unobtrusive monitoring technologies to foster integrative data collection across neural and behavioral dimensions
- Consider artificial gravity and individualized countermeasures as cornerstones for mitigating human spaceflight risks
- Promote data sharing and collaboration at all levels between researchers and agencies to leverage and maximize integrative research approach across disciplines

### Data sharing and international collaboration

To efficiently leverage such integrative approaches national and international collaborations between researchers and space agencies will be increasingly critical^103^. Overarching aspects linking to all scientific disciplines are clear. The inaccessibility of operational performance data to the scientific community complicates the study of the relationships between physiological changes and potential performance deficits (e.g., piloting performance, emergency egress, manual control, etc.) during critical mission phases. Protocols should be established for the disclosure of operational performance data to qualified researchers. Promoting closer partnerships among the scientific, operations, and training communities could be an efficient step to pave this way. Furthermore, the development of a data repository archiving past experimental data sets and information on their contexts could be very valuable to enhance the usability of previous data (e.g., challenge the low-N problem in space research).

## Benefit for earth and industrial relevance

Understanding how the brain adapts to spaceflight and processes gravitational information to shape actions is a fundamental yet challenging question. Every step toward this ambitious objective will open new doors to unexplored arenas, all of which with the potential to bring innovation transferrable to terrestrial applications. Mobile miniaturized monitoring technologies developed for spaceflight could foster the unobtrusive collection of neurophysiological data to detect adverse neurobehavioral outcomes at an early stage, and potentially avoid clinical manifestations in response to environmental conditions, psychological stressors, and unhealthy life-styles. Spaceflight analogs provide unique settings that promote novel insights into brain and behavioral plasticity on Earth^104^. Bed rest or dry immersion can be considered models of aging. Likewise, isolation studies can serve as models of social distancing associated with pandemics. Changes that typically evolve over years can be mimicked in time-lapse, providing unique opportunities to explore aging-related diseases and their prevention. In addition, the high standardization and control of these studies (e.g., nutrition, sleep/wake cycles, and social activities) could promote the understanding of causal relationships between inactivity, brain function, and cognitive alterations. Collectively, these characteristics can translate the knowledge gained from these studies to the prevention and treatment of various clinical conditions associated with cognitive impairments, for which reduced physical activity levels are a critical risk factor such as chronic heart failure, Type II diabetes, obesity, myotonic dystrophy, fibromyalgia, various types of cancer, depression, anxiety, and dementia.

In addition to better understanding such phenomena and their mechanisms spaceflight research can also boost innovative and efficient technologies for monitoring and predicting adverse neurobehavioral conditions, and promote the development of innovative treatment strategies^105^. Technologies such as augmented feedback and virtual reality—also in combination with physical exercise—can help patients to accelerate recovery or serve as a training tool for promoting brain health^106^. Given the fundamental role of gravity on the human body the use of artificial gravity may open new avenues for promoting health and disease. In addition to short bouts of artificial gravity induces by short-arm human centrifuges, large rotating habitats that can house subjects for days could serve to better understand the effects of artificial gravity on the human organism, and potentially probe new interventions for modern epidemic health issues such as obesity, osteoporosis, sarcopenia, and cardiovascular deconditioning.

## Future outlook and summary

As space-faring nations across the globe are fueling a new race of human space exploration that goes well beyond the Moon, national agencies and private entities across the globe have accelerated the research and development that will promote the safety and success of such missions. These missions will be some of the most difficult, dangerous, and dynamic operations in history, and considerably longer than current standard missions on ISS. Under current and notional NASA plans, crewed orbital missions to Mars could exceed 1,000 days, and exploration campaigns could even span decades. Such prolonged mission durations could introduce potential unprecedented astronauts’ health and performance risks. Adverse behavioral conditions and psychiatric disorders are considered one of the most serious but also least understood risks during such long-duration space missions. The effects of extreme environmental conditions and psychological stressors on brain and behavior are currently not fully understood. Future work in this field should be characterized by an interdisciplinary approach that promotes collaborations across space agencies, considers sex as a biological variable, and integrates multimodal brain imaging, psychological and behavioral, neurovestibular, cardiovascular, omics, sleep, and circadian data. Such an approach could promote a holistic understanding of intellectual frameworks that together exceed individual disciplinary perspectives. The knowledge from such approaches could go beyond their application to spaceflight and translate to the prevention and treatment of various clinical conditions on Earth including but not limited to neurogenerative diseases.
